# Book Review

**DOI:** 10.1155/1991/90241

**Published:** 1991

**Authors:** Bengt Jeppsson

**Affiliations:** Lund Sweden


					HPB Surgery, 1991, Vol. 3, p. 209
Reprints available directly from the publisher
Photocopying permitted by license only
(C) 1991 Harwood Academic Publishers GmbH
Printed in the United Kingdom
BOOK REVIEW
G Gozzetti, A Mazziotti, L Bolondi, L Barbara Intraoperative Ultrasonography in
Hepato-Biliary and Pancreatic Surgery -A Practical Guide. Edited by Kluwer
Academic Publishers, Dordrecht, Boston, London, 1989 (183 pages) ISBN 0-7923-
0261-3
This book is truly a practical guide for anyone wanting to use the new technology of
intraoperative ulotrasonography. It is written by members from a European group
of pioneers in this field at the University of Bologna. The book starts with a chapter
on Physical Principles and Instrumentation and Exploration Technique and
Ultrasound Terminology. Thereafter follows Application in Hepatic Surgery,
Biliary Tract Surgery, Pancreatic Surgery and Surgery for Portal Hypertension and
Liver Transplantation.
The text is well written and concise and it is illustrated by high quality
ultrasonographic images. These are mostly explained by drawings as well as
indication of the position of the probe on the organ. This approach further justifies
the title A Practical Guide.
The book is written by four specialists, including both surgeons and gastroentero-
logists and I think this is a good example of how this new technique should be
explored, i.e. by the organ specialist irrespective of belonging to a profession. The
wide clinical application of this technique is illustrated plentiful in the book. This is
soon evident to the reader of the book. The book can be highly recommended to
any surgeon or physician who wants to be introduced into the use of ultrasonogra-
phy in upper GI disease and surgery.
Bengt Jeppsson
Lund, Sweden
(Accepted by S. Bengmark 18 August 1990)
209

				

